# Setting Priorities for a Provincial Adolescent and Young Adult Oncology Program

**DOI:** 10.3390/curroncol29060322

**Published:** 2022-06-01

**Authors:** Julian Surujballi, Grace Chan, Caron Strahlendorf, Amirrtha Srikanthan

**Affiliations:** 1 The Ottawa Hospital Cancer Centre, Ottawa, ON K1H 8L6, Canada; jsurujballi@toh.ca; 2 The Ottawa Hospital Research Institute, Ottawa, ON K1H 8L6, Canada; 3 Department of Medicine, University of Ottawa, Ottawa, ON K1N 6N5, Canada; 4 BC Children’s Hospital, Vancouver, BC V6H 3N1, Canada; gchan@cw.bc.ca (G.C.); cstrahlendorf@cw.bc.ca (C.S.); 5 Department of Medicine, The Ottawa Hospital, Ottawa, ON K1H 8L6, Canada

**Keywords:** AYA, oncology, program development

## Abstract

Adolescent and young adult (AYA, ages 15–39 years) oncology patients are an underserved population with specialized needs. AYA programs are absent from most Canadian centers. We identified a priority list and sequence for new programs to address. Program goals, priorities, and activities were developed through literature review, national consensus documents, and expert opinion. Health care providers (HCPs) involved in AYA cancer care, administrators, and patient and family representatives were engaged to co-develop program goals and activities. A modified Delphi technique was used through two iterations followed by an in-person meeting to prioritize program implementation. Consensus was defined as a mean score of less than 2.0 (not important) or 4.0 or greater (important). Items without consensus (scored between 2.0 and 3.99) were discussed at the in-person meeting. Sixty provincial stakeholders completed the Delphi survey across multiple disciplines. Twenty-seven stakeholders attended the in-person meeting. All goals were deemed important, except development of a research program. Patient implementation tasks ranked highest. Priority sequence of implementation was: patient care first, followed by HCP education; patient and family education; program sustainability plan; evaluation; research; then a model for multidisciplinary tumor board review. These represent key goals for new AYA oncology programs and a priority sequence of implementation.

## 1. Introduction

Adolescents and young adults (AYA, defined as ages 15–39 years old) with cancer suffer interruption of normative physical, behavioral, cognitive, and emotional development [1,2,3]. The AYA period includes development of values, personal identity, formation of strong personal relationships, starting families, and attaining financial independence [4,5]. A cancer diagnosis disrupts this development, whether through facing early death, interruption of social life activities, returning to live with parents, and/or fearing for the future due to treatment late effects or recurrence [4]. In addition, AYAs may experience more intense symptom burden, have less-developed coping mechanisms, and exhibit poorly developed autonomy in decision making [4]. Families of AYAs with cancer also experience distress, which may compromise their ability to support AYA patients. Although the most inclusive definition ranges from ages 15–39, programs worldwide vary in patient inclusion, depending on local resources and needs [3,6,7,8]. 

Cancer is the leading cause of disease-related death in adolescents and young adults (AYAs) in the US and Canada [9,10]. Despite improving survival among the broader AYA cancer population, survival rates continue to lag behind those observed in younger and older populations for specific cancer types, such as breast cancer, and sarcoma [6,11]. There are deficiencies for AYA in care across the cancer journey, through diagnosis and treatment, to survivorship or palliative care. Multiple factors impact this disparity, including diagnostic delay, more aggressive disease biology, poor treatment adherence, and system issues such as poor processes and structures to address unique AYA needs [12,13,14,15]. 

Recognizing the uniqueness of this population, current recommendations state that AYA cancer therapy be administered by individuals with AYA-specific expertise [16,17]. Despite these recommendations, many oncology programs in Canada lack a dedicated AYA program, and those that exist lack standardization. Thus, AYAs aged 15–21 years may thus receive care in pediatric or adult systems, although neither system is specifically designed for the specific needs of this vulnerable group [18]. This provides the opportunity to design new bespoke programs that meet the needs of health care providers (HCPs), patients, and families specific to the Canadian context. Co-designing programs that meet the needs of all end-users requires involvement of all affected parties, including patients, families, health care providers, and health care administrators. Though AYA programs have been proposed in the past, limited data exist regarding implementation sequencing at the ground level. To that end, we describe the efforts undertaken in the province of British Columbia (BC), Canada to identify the key priorities for patients, families, front-line HCPs, and administrators with and without AYA expertise, in improving AYA patient care delivery. The goal of this work is to identify how various components of an AYA program may be best implemented and in which priority. 

For the context of this study, health care in Canada is largely delivered at the provincial level with rules, regulations, funding, and organization differing from province to province. Funding and oversight are provided by provincial organizations, such as BC Cancer in BC, to regional institutions where health care is delivered to patients. Larger academic centers typically receive more provincial funding and more staff that could be allocated to specific programs. As such, a provincial “umbrella” program is feasible through collaboration between provincial organizations and academic centers. Resources developed through this program could then be shared with regional centers. 

## 2. Materials and Methods

### 2.1. Study Design

Proposed AYA program goals, priorities, components, and activities (79 distinct items) were developed through literature review, national consensus documents, and provincial expert opinion (via the BC Cancer/BC Children’s provincial AYA Joint Steering Committee). A modified Delphi survey technique with two iterations was used to gather stakeholder input and feedback prior to a stakeholder meeting [19,20]. Consensus was defined as a mean score of less than 2.0 (indicating not important) or 4.0 or greater (indicating important). Items without consensus (scored between 2.0 and 3.99) after round one were discussed in-person by stakeholders. 

### 2.2. Program Components

The following program components were pre-identified by the BC AYA Joint Steering Committee. These components were: (1) program mission and goals; (2) patient care implementation; (3) health care provider (HCP) education strategy and needs assessment process; (4) patient and family education strategy; (5) program evaluation strategy; (6) model for multidisciplinary tumor boards; (7) model for program expansion and sustainability; and (8) AYA research priorities. For each component, program objectives, criteria, processes, and strategies were developed prior to the in-person stakeholder session. This was done using existing resources, expert opinion, national consensus, and peer-reviewed research (see Appendix B for a complete list of components). 

### 2.3. Participant Identification and In-Person Session Format

Key stakeholders involved in AYA cancer care from each health authority in BC were identified by contacting medical directors in each health authority, provincial heads of nursing, patient and family counseling, and pain and symptom management services. Participants who completed the online survey were invited to participate in the in-person session. Individuals were recruited for participation if they had at least 5 years of clinical oncology experience post terminal degree training, and 10% of their adult clinical practice included AYA cancer patients. For the health care provider participants based out of pediatric institutions, a percentage of AYA clinical practice was not pre-specified. Additionally, regional leaders who are aware of early-career staff recognized as AYA champions were provided the opportunity to put additional names forward. See Appendix D for the in-person session agenda. 

For the in-person meeting, participants were assigned to groups of 5–6 individuals. Groups were provided with discussion guides and first-round Delphi survey results and asked to discuss each component. Results of these discussions were summarized narratively. Due to the size of the small groups, the multidisciplinary conference tumor board review and AYA research priority components were not discussed at the stakeholder session, as these components were ranked lowest for prioritization.

### 2.4. Data Analysis

Descriptive statistics were generated for participant demographics and Delphi responses. Mean Delphi results were presented. Survey respondents were asked to prioritize program component implementation, ranking each component on a scale of 1 to 7 (first to last). The frequencies of participant rankings for each item were summed. Items were ranked according to weighted mean rankings from lowest to highest.

## 3. Results

### 3.1. Respondent Characteristics

A total of 100 participants were invited to participate. Sixty participants completed the Delphi survey. Twenty-seven individuals attended the in-person session. Appendix A (Table A1) provides demographic details on survey respondents and session participants. Respondents included administration (6.7%), patient and family representatives (1.7%), oncology physicians (26.7%), nursing (26.7%), counseling (21.7%), pain and symptom management (6.7%), psychiatry (1.7%), nurse practitioners (1.7%), speech–language pathology (1.7%), nutrition (1.7%), and unspecified (1.7%)

### 3.2. Delphi Survey and Round Table Discussion Results

**Figure 1:**
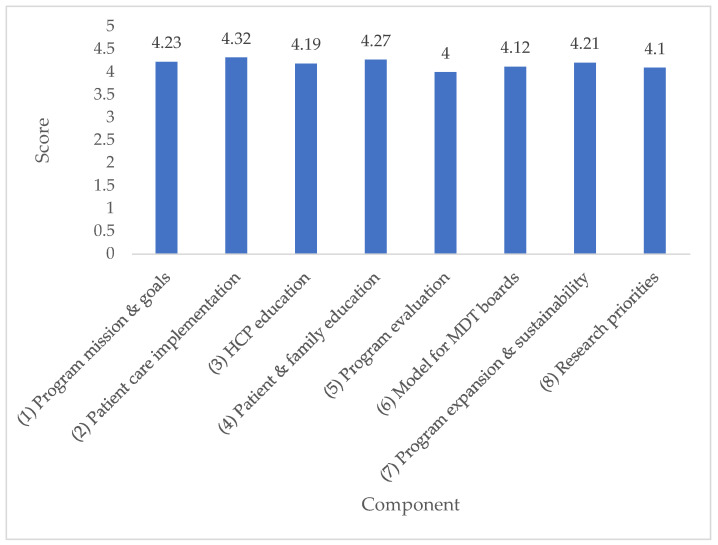
Average score per component.

**Figure 2:**
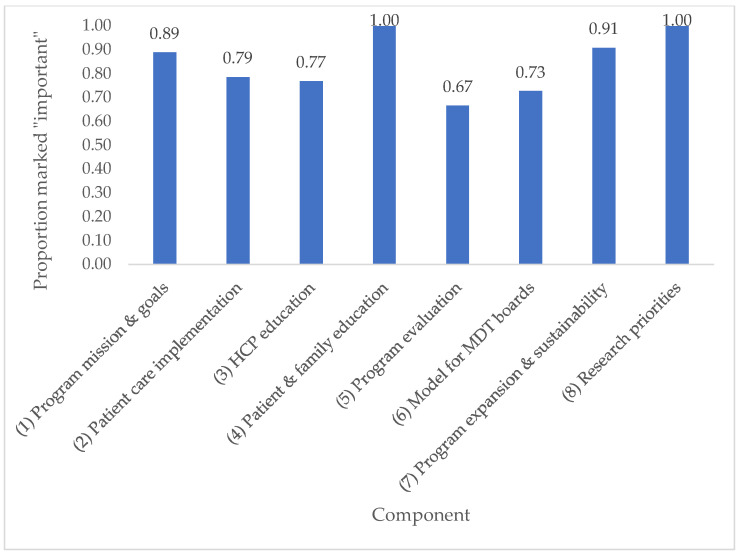
Proportion of items rated “important” per component.

After two rounds of the Delphi survey, consensus was reached on 84% of items. All items on which consensus was reached were deemed important. Full details of Delphi survey results and the discussion guide are available in Appendix B. The top 10 highest rated items across all components are listed in Table 1. Average scores per program component are shown in Figure 1 and the proportion of items rated “important” per component is shown in Figure 2. A complete list of items rated “important” is provided in Appendix C. 

All program goals were endorsed as important, except the development of an AYA research program. Priority of program implementation was ranked as patient care first, followed by: HCP education; patient and family education; research; program sustainability plan; evaluation; then model for multidisciplinary tumor board review. Of the various program activities, patient implementation tasks ranked highest. Common themes that emerged from table discussions during the in-person meeting are categorized and summarized narratively below.

#### 3.2.1. Scope of Program

Groups highlighted the importance of creating a provincial AYA program, with a provincial umbrella to provide consistent information, resources, and guidelines to regional programs (five of five groups). Regional centers should consider regional context and link to local resources (five of five groups). The need for integration of alternative ways of care delivery (such as telemedicine, or virtual care) to expand provincial reach was noted (three of five groups).

The age range of 15–29 years versus 39 years as the upper age limit was debated. The 15–29 age range was suggested for pilot programs, with increased eligibility to age 39 for a provincial program (five of five groups), with flexible age cutoffs depending on the tumor group. Program components thought to be missing from the original program goals include AYA survivorship integration (three of five groups), focused fertility resources (two of five groups), and focused palliative care resources (one of five groups).

Participants recommended having an AYA “champion” or clinical lead in each tumor group, bone marrow transplant programs, diagnostic imaging, fertility services, and palliative care services. Implementation of the program would vary based on regional resources, including human health resources, and local demand for services. Discussions suggested that the most sustainable and impactful model would be to develop AYA regional hubs in major urban centers with higher resource capacity (for example, in Vancouver through the BC Cancer—Vancouver Centre), and the development of local AYA champions in less resourced areas, or the provision of virtual services. As regional volumes grow, capacity for multiple AYA regional hubs could be developed throughout a province (for example, the Fraser Health Authority including Surrey could be targeted for AYA resource development). 

#### 3.2.2. Psychosocial Services

Groups recommended development of an AYA-specific distress screening, with regular distress screening throughout the care trajectory (four of five groups). Similarly, items pertaining to psychosocial services scored highly across all domains (Table 2). It was agreed that psychosocial wellbeing should be measured and tracked as a program evaluation strategy, though there was no consensus on what metrics should be evaluated.

#### 3.2.3. Care Pathways

Participants noted the importance of clearly defined care pathways, with identified contact points throughout the care trajectory (three of five groups). Definitions of what a care pathway entailed were not specified, with recognition that disease groups would likely have different needs. To assist AYA patients with system navigation, an AYA resource person could be appointed (two of five groups). One group suggested that psychosocial screening should be done initially and on an ongoing basis, with the first screening and assessment within 48 h of the first oncology appointment, and thereafter every 2 weeks during active medical care. 

#### 3.2.4. Role of AYA Team

There were mixed opinions as to whether an AYA clinical specialist (such as an advanced practice nurse (APN) or counselor) should provide direct patient care (two of five groups) versus helping existing providers to deliver AYA care (three of five groups). Suggested possible roles for the APN included staff education, direct patient care, building AYA program capacity, and survivorship care. The importance of using communications technology (email, patient portal, apps, virtual support groups) was highlighted by two of the five groups. All five groups throughout discussions mentioned the need for AYA-specific screening tools to help address the unique psychosocial needs of this group.

#### 3.2.5. Health Care Provider Education Delivery

The group suggested that HCP education delivery could include rounds, newsletters, emails, champions, modules (with dedicated time or incentives), general practitioner in oncology (GPO) training, nursing lunch and learns, and by adding resources to institutional websites. 

#### 3.2.6. Priorities for Education

Top priorities were HCP education, needs assessments and educating family physicians on the survivorship needs of AYA patients (including sending care plans to family physicians). It was agreed that creation of an AYA fellowship program should be a long-term priority and should not be included in the pilot.

Suggested priority topics for patient and family education included palliative care, sexuality and sexual health, vocational rehabilitation, returning to work or school, survivorship and late effects including psychosocial needs, and transitioning to a new normal. The group agreed that all resources developed should be evidence-based and supported by literature. Proposed education delivery methods included web-based, patient portal, podcasts, Facebook live, webinars, YouTube, and at the point of care. Communication could occur using social media and posters with AYA images, and ideally be interactive. Proposed methods for peer support delivery were online, face to face, through peer volunteers, and through local organizations. Peer support can be social in nature, psychoeducational, or focus on expressive arts. 

#### 3.2.7. Patients and Family Engagement

The group agreed that engagement should be on an ongoing basis and include asking patients and families for feedback, questionnaires and follow-up in individualized ways that are meaningful to the person, and communications from individual AYA programs. There was emphasis to ensure staff are informed regarding patient engagement strategies, and that the process to engage is transparent. In addition, patient and family involvement should be incorporated in program evaluation. 

#### 3.2.8. Program Expansion and Sustainability

The group suggested building an inventory of available resources and adjusting as needed over time, as well as developing a separate website and app. The team should provide education to build capacity within each center. An AYA peer navigator should be identified to support patients in navigating the system during active treatment and beyond. It was agreed that dedicated funding is needed for the AYA team. A sustainable program requires support to liaise with the community and community resources. One suggestion was that a patient and family advisory council with regional representation should provide input into the program.

#### 3.2.9. Implementation Prioritization

Eighteen of 27 participants completed this section of the survey. Of these, 15 participants ranked all seven items while three did not (see Appendix E for individual results). Priority of program implementation was ranked as patient care first, followed by: HCP education; patient and family education; program expansion and sustainability; evaluation; research; then model for multidisciplinary tumor board review. Of the various program activities, patient implementation tasks ranked highest. 

#### 3.2.10. Potential Program Tasks

Actual delivery of the program will vary regionally based on local constraints, resources, and patient volumes, and final implementation will need to be negotiated with regional and provincial leaders, and adapted over time. The recommendations provided by the stakeholders provide an overview of the principles that should be in place and priority targets for development and implementation. Example tasks that could be undertaken at the provincial and regional level for the top ten high priority items that were discussed are highlighted (Table 3). 

## 4. Discussion

AYA program development is of value to a wide range of stakeholders. Herein, we present the first provincial efforts of developing priorities for ground-level implementation. This work is transferrable to other jurisdictions, as the highest ranked program components and discussion points raised are relevant to other institutions. Of the program components, patient care implementation was ranked as the highest priority for stakeholders, followed by health care provider education. Implementation of a multidisciplinary tumor board ranked lowest. 

Based on round table discussion, while individual regional programs should be developed to suit the needs of each center, regional “umbrella” programs are required to ensure that information, resources, and guidelines are consistent. This model allows for sharing of limited resources between centers and increases consistency of care regardless of geographic location. While the groups recommended this “umbrella” program be developed at the provincial level, alternatively this can be done at a national level for certain items (such as standards of care and guidelines) to avoid duplication of work between provinces while still allowing regional centers to grow as per their unique needs.

Items relating to referrals, direct patient care, and psychosocial support scored higher than those relating to research, quality improvement, or formation of tumor board case reviews. These findings are logical in the context of a pilot AYA program as patients, families, and front-line HCPs are more likely to benefit from these tangible interventions. This is consistent with existing evidence that communication between AYA patients and their HCPs remains poor, and distress support remains inadequate [21,22,23]. Pilot AYA programs focusing on patient care and psychosocial support resonate with front-line staff and patients. This is consistent with grassroots clinics that have developed thus far in various jurisdictions including Toronto, Montreal, and Alberta. Fertility preservation screening and referral were identified as specific issues that could be easily targeted as initial steps. This is consistent with national and international priorities in the AYA population [2,4,5,16,17]. 

While items pertaining to QI, research, and indirect patient care such as holding tumor board discussions did not score as highly, many did still reach the threshold for consensus and were deemed important. As a strategy to prevent detraction of resources away from direct patient care and psychosocial support, implementation of items that do not have immediate impact on patient care can be deferred until an institution’s AYA program is more established. Items pertaining to patient care implementation scored the highest of items across all domains, comprising the top three highest rated items and five of the top 10. Items relating to psychosocial support, automatic referrals, and follow-up through the AYA program, APN, or counselor were especially highly rated. It must be acknowledged, however, that AYA patients with cancer are an understudied population and thus establishing a research program will be essential for the future of AYA treatment and survivorship. Even if not implemented immediately, establishing a research program should be part of any AYA program and cannot be forgotten.

Despite consensus to create an AYA APN and counselor with whom patients would be offered consultation and follow-up, there was lack of consensus regarding their workflow. This represents the variation in needs of individual institutions even within similar jurisdictions. Recommendations ranged from the AYA staff seeing all AYA patients, instituting a referral-based process for high risk AYA, to no direct patient care responsibilities and capacity building among front-line staff alone. The majority felt some direct patient care would be beneficial, particularly for higher needs patients. In addition, the need to identify an AYA resource clinician with clinical expertise in AYA cancer care at each cancer center to support high needs patients, in addition to the AYA-specific APN and counselor, was identified. There is need for further clarification of these roles at the level of both umbrella and institutional program levels.

Although the average rating of items under HCP education was not as high as in other domains, the number of important items (10) was second highest, behind only patient care implementation (11). Based on consensus, completing HCP needs assessment surveys including the following topics is recommended: survivorship and late effects for AYA, the unique psychosocial needs of AYA, navigating interpersonal relationships for patients in treatment, palliative care needs for AYA, and coaching lifestyle changes, healthy diet, and exercise for AYA patients on treatment. These needs assessments would serve to ask HCPs what they need to succeed, in addition to providing evidence-based information on best practices in AYA oncologic care. This could include annual grand rounds on AYA oncology, development of online modules, and establishing partnerships with other organizations.

There was unanimous consensus for a strong focus on HCP education and capacity building, regardless of the future direct patient care role of the AYA-specific APN and counselor. Although patient care implementation was identified as the highest priority, HCP education is inherent to the provision of patient care [24] and requires less infrastructure to begin. Moving forward with HCP education either as an initial step or concurrently with patient care implementation based on each center’s resources will impact patient care. Focusing on HCP education will improve direct patient care through existing personnel and staff by improving knowledge and skill sets. By providing such continuing education opportunities, each program will also conform with best practices to ensure ongoing development of staff skills. It was evident that staff and patient and family partners recognize that providing care to this demographic is challenging, particularly when raising distressing topics, such as loss of fertility, ongoing and long-term toxicity, and incurable diagnoses. Providing direct support to HCPs and patients during these higher stress interactions will improve delivery of care and improve HCP and patient satisfaction.

Existing AYA clinical programs exist to varying degrees in Canada. In Toronto, for example, the Princess Margaret Cancer Centre provides a local AYA program which patients and health care providers can refer into. With this program, a clinical nurse specialist provides counseling, and referrals and direction to various resources in the tertiary centers and community that would be relevant to the individual patient’s concerns [25]. Alberta Health Services (AHS) is another example. The AHS program provides AYA patient navigators, who are specially trained registered nurses, at the Edmonton and Calgary cancer sites to provide individualized support to patients, facilitate referrals to appropriate services, and link patients to available resources [26]. Although differing in regional scope, both programs prioritized clinical delivery of care through AYA-specific health care providers who can help navigate health care systems and provide direction towards psychosocial support. The current study identifies how to further expand on existing programs by suggesting proposed next steps for implementation of more comprehensive AYA programs.

### Limitations

This work has limitations. Despite initial invitations, limited responses from patient and family partners were received during the online iterations. However, despite this set-back, the overall findings are consistent with national guidelines which were developed with patient and family representatives and feedback from national AYA advocacy groups. As the primary goal was to develop an implementation strategy within local centres, diverse feedback from front-line clinicians, administrators, and clinicians in managerial roles was needed and successfully obtained.

## 5. Conclusions

Improving AYA delivery of care is an important priority for stakeholders. This body of work provides practical steps to support cancer centers in the development of local programs. Recruiting AYA clinicians to develop and deliver programs and improving health care provider competencies through education endeavors serves as the initial next step institutions can undertake.

## Figures and Tables

**Table 1:** Top 10 highest rated items across all components.

Domain	Score	Description
Patient care implementation	4.67	Establish referral pathways for pre-defined high problem issues (such as suicide, psychosocial distress, fertility preservation, urgent end of life symptom management)
Patient care implementation	4.66	Develop referral pathways for patients referred to BC Cancer, BCCH, VGH based on age and diagnosis
Patient care implementation	4.64	Create process so all AYA patients offered AYA program consultation with advanced practice nurse (APN) and counselor
Program mission and goals	4.61	Program mission: create a provincial interdisciplinary cancer program for AYA aged 15–29 years that will regionally implement recommendations across all BC Cancer sites in partnership with BCCH
Patient care implementation	4.57	Create process so all AYA patients offered follow-up with APN or counselor during treatment trajectory
Program expansion and sustainability	4.56	Foster relationships with motivated survivors, patients, and families for ongoing advocacy
Patient care implementation	4.53	Ensure all AYA patients screened for distress at intake
HCP education	4.53	HCP needs assessment survey topic: the unique psychosocial needs of AYA
Patient and family education	4.5	Develop patient education materials on survivorship and late effects for AYA
Patient and family education	4.48	Develop patient education materials on fertility preservation and counseling

**Table 2:** Item ratings relating to psychosocial services across all program components.

Score	Program Item	Component
4.67	Establish pathways for high problem issues (such as suicide, psychosocial distress, fertility preservation, urgent end of life symptom management)	Patient Care
4.66	Create referral pathways at each institution based on age and diagnosis	Patient Care
4.64	Ensure all AYA patients offered AYA program consultation with advanced practice nurse (APN) and counselor	Patient Care
4.57	Create process so all AYA patients offered follow-up with APN or counselor during treatment trajectory	Patient Care
4.56	Foster relationships with motivated survivors, patients, and families for ongoing advocacy	Sustainability
4.53	HCP education topic: the unique psychosocial needs of AYA	HCP Education
4.50	Patient education materials on survivorship and late effects for AYA	Patient/Family Education

**Table 3:** Example tasks of the proposed AYA program.

Domain Priority	Description of Priority Item	Provincial Task	Regional Task
Patient care implementation	Establish referral pathways for pre-defined high problem issues (such as suicide, psychosocial distress, fertility preservation, urgent end of life symptom management)	Establish provincial working group to create best practice standard operating procedures, including appropriate screening tools and timelines for access to care	Identify available local resources
			Establish locations for alternate care when local resources lacking
	Develop referral pathways for patients referred to different locations based on age and diagnosis		Clarify and establish local limitations through process mapping
			Establish human resource targets for optimal staffing
	Create process so all AYA patients offered AYA program consultation with advanced practice nurse (APN) and counselor	Develop human resource job descriptions for AYA program staff and establish number of staff needed per population	Determine if referral will happen at new patient registration or after first consultation
	Create process so all AYA patients offered follow-up with APN or counselor during treatment trajectory	Create standards for timelines to referral, and frequency of follow-up assessments based on disease site	Automate and deliver routine screening for AYA-specific distress factors
	Ensure all AYA patients screened for distress at intake		
Health care provider education	HCP needs assessment survey topic: the unique psychosocial needs of AYA	Establish funding and education opportunities for various AYA HCPs	Create local standards for continuing education opportunities
Patient and family education	Develop patient education materials on survivorship and late effects for AYA	Create electronic and written resources	Tailor individual education to patient needs by front-line staff
	Develop patient education materials on fertility preservation and counseling	Provide easily identifiable mechanism for navigating to resource (i.e., provincial website)	Local referrals to appropriate regional centers
Program expansion and sustainability	Foster relationships with motivated survivors, patients, and families for ongoing advocacy	Support and maintain patient and family advisory mechanisms	Promote engagement among motivated AYA patients and family
		Identify and recruit ongoing participants specific to AYA cancer	Direct individuals to available opportunities

## Data Availability

The data presented in this study are available in this article.

## Appendix A

**Table A1:** Delphi survey respondent and engagement session participant areas of work.

	Round 1		Round 2	
Area of Work	Responses (*n*)	%	Participants (*n*)	%
Administration	4	6.7%	1	3.7%
Oncology	16	26.7%	10	37.0%
Nursing	16	26.7%	4	14.8%
Psychosocial and Therapeutic Services	13	21.7%	5	18.5%
Radiation Therapy	1	1.7%	0	0%
Pain and Symptom Management	4	6.7%	0	0%
Nutrition	1	1.7%	0	0%
Patient/Caregiver Advisor	1	1.7%	3	11.1%
Psychiatry	1	1.7%	1	3.7%
Nurse Practitioner	1	1.7%	1	3.7%
Speech Language Pathology	1	1.7%	0	0%
Vocational Rehabilitation	0	0%	1	3.7%
Other—Patient Advocate	0	0%	1	3.7%
No Response	1	1.7%	0	0%
Total	60	100%	27	100%

## Appendix B

Note: those items scored <2.00 or >3.99 in round one of the survey were interpreted as either unimportant and excluded from program development, or important and included in program development. Those items that scored from 2.01–3.99 were interpreted as inconclusive, and rescored in the second survey iteration. The table below shows the weighted mean for rounds one and two of the survey. Those items in yellow in round one were inconclusive and rescored, and, if green in round two, are important and should be included in the program.

### Appendix B.1. Discussion Guides

**Program Mission and Goals—Discussion Guide**
Program mission: Create a provincial interdisciplinary cancer program for adolescents and young adults aged 15–29 years that will regionally implement recommendations across all BC Cancer sites in partnership with BC Children’s Hospital - Is this mission appropriate? - Is the age range appropriate? - Are there others who should be involved? Review program goals with the following questions in mind: - Are these goals comprehensive enough for a provincial program? - Is there anything missing? Anything that should be omitted? - What are the priority goals for implementation?

**Patient Care Implementation—Discussion Guide**
- Should all AYA patients receive a consult with AYA program advanced practice nurse (APN) or counselor? If not, what are the ideal consultation criteria? - The new AYA program will hire an APN and counselor to take on the following activities/roles: ○ Introduce AYA program to patients and families ○ Administer AYA distress screen to identify immediate needs ○ Patient and family counseling and education ○ Provide access to AYA specific information ○ Facilitate referral to speciality services ○ Fertility counseling and referral ○ Links to community resources ○ Links to family counseling and support services ▪ Based on the above, are there any additional activities the APN and counselor should engage in with patients and families? - Should the APN or counselor see patients during existing oncology appointments? - How frequently should APN or counselor check in with patients throughout treatment trajectory and post treatment? - What AYA-specific screening tools are needed? - What role should the AYA APN and counselor play in capacity building of BC Cancer staff?

**Health Care Provider Education—Discussion Guide**
- Is the list of education topics exhaustive? Are there other education topics that should be included? - How can health care provider education be best delivered? - How can primary care providers be involved in AYA oncology education? - What would partnerships with various organizations, such as FPON, CON sites, Divisions of Family Practice, look like for health care provider education?

**Program Evaluation Strategy—Discussion Guide**
- How can we collect patient reported outcomes from AYA patients across all tumor groups? - Review the proposed measures in the draft evaluation strategy—are these the right things to collect? - How should evaluation updates be shared with patients, families, and senior leadership on a regular basis?

**Patient and Family Education and Engagement—Discussion Guide**
- Review the list of patient education material topics in the Delphi survey below. Is this list exhaustive? Are other topics needed? - What is the best way to provide patient and family education? (e.g., printed materials, new AYA website, etc.) - What would an AYA peer support network look like for BC? - How can we engage patients and families on an ongoing basis to inform program development?

**Program Expansion and Sustainability—Discussion Guide**
- What is the best way to expand the program? (for example, increase age range to 39; have AYA specialist APNs/counselors at each center; capacity building with existing BC Cancer staff and have AYA APN and counselor as specialists available for consultation) - What needs to be in place to sustain this program? - What is currently feasible at each center?

### Appendix B.2. Delphi Survey Results Summary

**Table:**

Item	Round 1 Weighted Mean	Round 2 Weighted Mean
Program Mission and Goals		
Program mission: Create a provincial interdisciplinary cancer program for adolescents and young adults aged 15–29 years that will regionally implement recommendations across all BC Cancer sites in partnership with BCCH	**4.61**	-
Goal 1: Develop health care provider education curriculum based on a formal learning needs assessment with clinical teams	**4.08**	-
Goal 2: Facilitate clinical consults with referral pathways to other services and flexible access to interventions	**4.45**	-
Goal 3: Integrate access to AYA-specific psychosocial distress screening and fertility preservation screening and referral	**4.41**	-
Goal 4: Develop multidisciplinary tumor board case reviews supported at BCCH and BC Cancer	3.78	**4.04**
Goal 5: Create evidence-based quality improvement and program evaluation plans	**4.02**	-
Goal 6: Develop a comprehensive AYA research program	3.83	**3.81**
Goal 7: Introduce patient reported outcome measurement to improve patient experience	**4.18**	-
Goal 8: Support patients and families through education and peer support network	**4.43**	-
Patient Care Implementation		
Develop referral pathways for patients referred to BC Cancer, BCCH, VGH based on age and diagnosis	**4.66**	-
Ensure all AYA patients screened for distress at intake	**4.53**	-
Create process so all AYA patients offered AYA program consultation with advanced practice nurse (APN) and counselor	**4.64**	-
Create process so all AYA patients offered follow-up with APN or counselor during treatment trajectory	**4.57**	-
Create process so all AYA patients routinely contacted at pre-specified times during care trajectory for follow-up of clinical issues	**4.30**	-
AYA APN and/or counselor to see patients referred into the program only (referral-based program)	3.98	3.91
AYA APN and/or counselor meet with all AYA patients at least once after new patient intake appointment	**4.38**	-
AYA APN and counselor to work out of a dedicated space separate from current oncology clinics	3.15	3.70
AYA APN and counselor to see patients during pre-existing oncology clinic appointments	3.79	3.96
Develop/adapt and implement AYA-specific screening tools (psychological distress, fertility screening)	**4.33**	-
Establish referral pathways for pre-defined high problem issues (such as suicide, psychosocial distress, fertility preservation, urgent end of life symptom management)	**4.67**	-
Establish transition pathways between BCCH, BC Cancer, and VGH	**4.45**	-
Develop/adapt tumor-specific treatment guidelines	**4.05**	-
Develop/adapt AYA-specific supportive care guidelines	**4.35**	-
Health Care Provider Education Strategy		
Conduct health care provider (HCP) education needs assessment through surveys of nursing, counseling, and physicians at BCCH and BC Cancer asking respondents to rank education priorities	3.98	3.79
Conduct education needs assessment among GPOs via survey asking respondents to rank education priorities	3.23	**4.04**
Conduct education needs assessment among primary care providers via survey asking respondents to rank education priorities	3.88	
HCP needs assessment survey topic: fertility preservation and counseling	**4.40**	-
HCP needs assessment survey topic: survivorship and late effects for AYA	**4.44**	-
HCP needs assessment survey topic: the unique psychosocial needs of AYA	**4.53**	-
HCP needs assessment survey topic: navigating interpersonal relationships for patients in treatment	**4.38**	-
HCP needs assessment survey topic: palliative care needs for AYA	**4.47**	-
HCP needs assessment survey topic: coaching lifestyle changes, healthy diet, and exercise for AYA patients on treatment	**4.20**	-
Development of online continuing medical education accredited module in AYA oncology targeted to all health care providers involved in AYA care	3.91	**4.13**
Development of AYA fellowship program with Royal College diploma for Focused Competency in Adolescent and Young Adult Oncology	3.80	3.67
Annual grand rounds on AYA oncology	3.65	**4.42**
Establish formal partnerships between various organizations for ongoing HCP education (i.e., Family Practice Oncology Network, Community Oncology Network sites)	3.96	**4.09**
Patient and Family Education and Engagement Strategy		
Conduct environmental scan to determine top educational needs of AYA patients and families	**4.38**	-
Conduct environmental scan to identify existing AYA patient and family education resources to be adapted to BC context	**4.05**	-
Gather BC Cancer and BCCH new patient materials to adapt to AYA needs	**4.06**	-
Develop patient education materials on fertility preservation and counseling	**4.48**	-
Develop patient education materials on survivorship and late effects for AYA	**4.50**	-
Develop patient education materials on healthy lifestyle including nutrition and exercise	**4.24**	-
Develop an AYA peer support network	**4.30**	-
Develop a long-term patient and family engagement strategy for ongoing feedback into program development	**4.14**	-
Program Evaluation Strategy		
Ensure collection of adequate baseline outputs and outcomes prior to program implementation	**4.00**	-
Identify opportunities for data collection within existing resources	**4.00**	-
Identify additional resources required (including staff) for ongoing data collection	3.95	**4.23**
Establish frequency of data collection and frequency of review by oversight committee	3.85	3.73
Establish outputs and outcomes of relevance for data collection	**4.24**	-
Determine how evaluation updates will be shared with patients, family, and senior leadership on a regular basis	3.86	3.82
Model for Multidisciplinary Conference (MDC) Tumor Board Review		
Identify required team members for tumor board attendance and optional attendees	3.79	**4.05**
Representatives from medical oncology, radiation oncology, surgery/surgical oncology, pathology, diagnostic radiology, nursing, and patient and family counseling should be present to provide the complete range of expert opinion appropriate for the disease site and appropriate for the hospital	**4.45**	-
Representatives for BCCH and BC Cancer oncology services will attend conference	**4.23**	-
Establish ideal frequency, date, and timing of tumor boards and how notification will be undertaken—MDC should occur for a minimum of 1 h every 2 weeks	3.82	3.89
Identify electronic/online tumor board opportunities and platforms	**4.21**	-
All new AYA patient treatment plans should be forwarded to AYA MDC coordinator	**4.04**	-
Not all cases forwarded to the MDC coordinator need to be discussed at the AYA MDC	3.82	3.80
The individual physician and the MDC chair can determine which cases are discussed in detail at the MDC	3.94	**4.15**
Other cases (e.g., recurrent or metastatic cancer) can be forwarded to the MDC coordinator for discussion, at the discretion of the individual physician	3.89	3.80
AYA MDC will primarily serve to identify all suitable treatment options, and ensure the most appropriate treatment recommendations are generated for each cancer patient discussed prospectively in a multidisciplinary forum	**4.43**	-
Secondary functions of AYA MDC will include: a forum for the continuing education of medical staff and health professionals, contributing to the development of standardized patient management protocols, and contributing to linkages among regions to ensure appropriate referrals and timely consultation	**4.22**	-
AYA Research Priorities		
Form an AYA research and evaluation working group	**4.15**	-
Identify platforms for data collection	**4.05**	-
Develop and implement a program evaluation strategy through research	**4.00**	-
Identify patient reported outcomes and clinical outcomes for collection	**4.29**	-
Develop a quality improvement plan and strategy	**4.14**	-
Identify key stakeholders for AYA research	**4.00**	-
Identify and implement an AYA research agenda	3.95	**4.05**
Develop an AYA research education plan (e.g., fellowship training for HCPs)	3.76	**4.10**
Model for Program Expansion and Sustainability		
Identify key clinical and operations stakeholders for ongoing expansion	**4.23**	-
Develop website content and create a program email address	**4.42**	-
Develop social media strategies	**4.00**	-
Foster and develop online platform and app development	**4.26**	-
Review different models of expansion (spoke and hub; health authority-specific champions)	**4.11**	-
Identify available resources for de-centralized telemedicine expansion capacity	**4.09**	-
Identify available resources (including HCP compensation) for physical expansion capacity	**4.13**	-
Identify and explore operations and infrastructure limitations to expanding AYA program	3.84	**4.38**
Identify BC Cancer Foundation long-term funding opportunities	**4.49**	-
Identify AYA “champions” in regional centers to for ongoing program development	**4.29**	-
Foster relationships with motivated survivors, patients, and families for ongoing advocacy	**4.56**	-

Bold indicates final score.

## Appendix C. All Items Rated Important across All Domains

**Table:**

Program Item	Score	Component
Program mission: Create a provincial interdisciplinary cancer program for adolescents and young adults aged 15–29 years that will regionally implement recommendations across all BC Cancer sites in partnership with BCCH	4.61	Program Mission and Goals
Goal 1: Develop health care provider education curriculum based on a formal learning needs assessment with clinical teams	4.08	Program Mission and Goals
Goal 2: Facilitate clinical consults with referral pathways to other services and flexible access to interventions	4.45	Program Mission and Goals
Goal 3: Integrate access to AYA-specific psychosocial distress screening and fertility preservation screening and referral	4.41	Program Mission and Goals
Goal 4: Develop multidisciplinary tumor board case reviews supported at BCCH and BC Cancer	4.04	Program Mission and Goals
Goal 5: Create evidence-based quality improvement and program evaluation plans	4.02	Program Mission and Goals
Goal 7: Introduce patient reported outcome measurement to improve patient experience	4.18	Program Mission and Goals
Goal 8: Support patients and families through education and peer support network	4.43	Program Mission and Goals
Develop referral pathways for patients referred to BC Cancer, BCCH, VGH based on age and diagnosis	4.66	Patient Care Implementation
Ensure all AYA patients screened for distress at intake	4.53	Patient Care Implementation
Create process so all AYA patients offered AYA program consultation with advanced practice nurse (APN) and counselor	4.64	Patient Care Implementation
Create process so all AYA patients offered follow-up with APN or counselor during treatment trajectory	4.57	Patient Care Implementation
Create process so all AYA patients routinely contacted at pre-specified times during care trajectory for follow-up of clinical issues	4.30	Patient Care Implementation
AYA APN and/or counselor meet with all AYA patients at least once after new patient intake appointment	4.38	Patient Care Implementation
Develop/adapt and implement AYA-specific screening tools (psychological distress, fertility screening)	4.33	Patient Care Implementation
Establish referral pathways for pre-defined high problem issues (such as suicide, psychosocial distress, fertility preservation, urgent end of life symptom management)	4.67	Patient Care Implementation
Establish transition pathways between BCCH, BC Cancer, and VGH	4.45	Patient Care Implementation
Develop/adapt tumor-specific treatment guidelines	4.05	Patient Care Implementation
Develop/adapt AYA-specific supportive care guidelines	4.35	Patient Care Implementation
Conduct education needs assessment among GPOs via survey asking respondents to rank education priorities	4.04	Health Care Provider Education Strategy
HCP needs assessment survey topic: fertility preservation and counseling	4.40	Health Care Provider Education Strategy
HCP needs assessment survey topic: survivorship and late effects for AYA	4.44	Health Care Provider Education Strategy
HCP needs assessment survey topic: the unique psychosocial needs of AYA	4.53	Health Care Provider Education Strategy
HCP needs assessment survey topic: navigating interpersonal relationships for patients in treatment	4.38	Health Care Provider Education Strategy
HCP needs assessment survey topic: palliative care needs for AYA	4.47	Health Care Provider Education Strategy
HCP needs assessment survey topic: coaching lifestyle changes, healthy diet, and exercise for AYA patients on treatment	4.20	Health Care Provider Education Strategy
Development of online continuing medical education accredited module in AYA oncology targeted to all health care providers involved in AYA care	4.13	Health Care Provider Education Strategy
Annual grand rounds on AYA oncology	4.42	Health Care Provider Education Strategy
Establish formal partnerships between various organizations for ongoing HCP education (i.e., Family Practice Oncology Network, Community Oncology Network sites)	4.09	Health Care Provider Education Strategy
Conduct environmental scan to determine top educational needs of AYA patients and families	4.38	Patient and Family Education and Engagement Strategy
Conduct environmental scan to identify existing AYA patient and family education resources to be adapted to BC context	4.05	Patient and Family Education and Engagement Strategy
Gather BC Cancer and BCCH new patient materials to adapt to AYA needs	4.06	Patient and Family Education and Engagement Strategy
Develop patient education materials on fertility preservation and counseling	4.48	Patient and Family Education and Engagement Strategy
Develop patient education materials on survivorship and late effects for AYA	4.50	Patient and Family Education and Engagement Strategy
Develop patient education materials on healthy lifestyle including nutrition and exercise	4.24	Patient and Family Education and Engagement Strategy
Develop an AYA peer support network	4.30	Patient and Family Education and Engagement Strategy
Develop a long-term patient and family engagement strategy for ongoing feedback into program development	4.14	Patient and Family Education and Engagement Strategy
Ensure collection of adequate baseline outputs and outcomes prior to program implementation	4.00	Program Evaluation Strategy
Identify opportunities for data collection within existing resources	4.00	Program Evaluation Strategy
Identify additional resources required (including staff) for ongoing data collection	4.23	Program Evaluation Strategy
Establish outputs and outcomes of relevance for data collection	4.24	Program Evaluation Strategy
Identify required team members for tumor board attendance and optional attendees	4.05	MDC Tumor Board Review
Representatives from medical oncology, radiation oncology, surgery/surgical oncology, pathology, diagnostic radiology, nursing, and patient and family counseling should be present to provide the complete range of expert opinion appropriate for the disease site and appropriate for the hospital	4.45	MDC Tumor Board Review
Representatives for BCCH and BC Cancer oncology services will attend conference	4.23	MDC Tumor Board Review
Identify electronic/online tumor board opportunities and platforms	4.21	MDC Tumor Board Review
All new AYA patient treatment plans should be forwarded to AYA MDC coordinator	4.04	MDC Tumor Board Review
The individual physician and the MDC chair can determine which cases are discussed in detail at the MDC	4.15	MDC Tumor Board Review
AYA MDC will primarily serve to identify all suitable treatment options, and ensure the most appropriate treatment recommendations are generated for each cancer patient discussed prospectively in a multidisciplinary forum	4.43	MDC Tumor Board Review
Secondary functions of AYA MDC will include: a forum for the continuing education of medical staff and health professionals, contributing to the development of standardized patient management protocols, and contributing to linkages among regions to ensure appropriate referrals and timely consultation	4.22	MDC Tumor Board Review
Form an AYA research and evaluation working group	4.15	AYA Research Priorities
Identify platforms for data collection	4.05	AYA Research Priorities
Develop and implement a program evaluation strategy through research	4.00	AYA Research Priorities
Identify patient reported outcomes and clinical outcomes for collection	4.29	AYA Research Priorities
Develop a quality improvement plan and strategy	4.14	AYA Research Priorities
Identify key stakeholders for AYA research	4.00	AYA Research Priorities
Identify and implement an AYA research agenda	4.05	AYA Research Priorities
Develop an AYA research education plan (e.g., fellowship training for HCPs)	4.10	AYA Research Priorities
Identify key clinical and operations stakeholders for ongoing expansion	4.23	Model for Program Expansion and Sustainability
Develop website content and create a program email address	4.42	Model for Program Expansion and Sustainability
Develop social media strategies	4.00	Model for Program Expansion and Sustainability
Foster and develop online platform and app development	4.26	Model for Program Expansion and Sustainability
Review different models of expansion (spoke and hub; health authority-specific champions)	4.11	Model for Program Expansion and Sustainability
Identify available resources for de-centralized telemedicine expansion capacity	4.09	Model for Program Expansion and Sustainability
Identify available resources (including HCP compensation) for physical expansion capacity	4.13	Model for Program Expansion and Sustainability
Identify and explore operations and infrastructure limitations to expanding AYA program	4.38	Model for Program Expansion and Sustainability
Identify BC Cancer Foundation long-term funding opportunities	4.49	Model for Program Expansion and Sustainability
Identify AYA “champions” in regional centers to for ongoing program development	4.29	Model for Program Expansion and Sustainability
Foster relationships with motivated survivors, patients, and families for ongoing advocacy	4.56	Model for Program Expansion and Sustainability

## Appendix D. Agenda

BC Adolescent and Young Adult Oncology Program
Create the Program You Need

**Table:**

Time	Item Number	Items
8:30 a.m.	1.0	Registration, Coffee & Continental Breakfast
9:00 a.m.	2.0	Welcome & Opening Remarks
	2.1	A Family’s Experience with AYA Cancer Care
	2.2	Cancer Care for AYA in BC—Current Situation
	2.3	BC AYA Oncology Program—Program Development to Date
10:00 a.m.	3.0	Table Discussion & Report Back: Program Mission & Goals
11:30 a.m.	-Lunch-	
12:30 p.m.	4.0	Table Discussion & Report Back: Patient Care Implementation
2:30 p.m.	-Break-	
3:00 p.m.	5.0	Breakout Sessions
	5.1	Health Care Provider Education Strategy
	5.2	Patient & Family Education Strategy
	5.3	Program Evaluation Strategy
	5.4	Model for Multidisciplinary Tumour Review Board
	5.5	Program Sustainability & Expansion Plan
4:30 p.m.	6.0	Breakout Session Report Back & Large Group Discussion
5:00 p.m.	7.0	Closing Remarks & Next Steps

## Appendix E. Program Implementation Prioritization—Individual Results

**Table:**

	Health Care Provider Education	Patient and Family Education	Patient Care Implementation	Program Expansion and Sustainability	Research Priorities	MDC Tumor Board Review	Evaluation Strategy
Respondent				Rank in order of prioritization 1–7			
1	-	-	-	1	-	3	2
2	3	2	1	6	4	5	7
3	4	5	1	3	2	6	7
4	2	1	3	4	6	7	5
5	2	3	1	4	5	-	6
6	3	2	1	-	-	4	-
7	2	1	3	4	5	6	7
8	3	1	2	5	4	7	6
9	1	2	3	7	4	5	6
10	3	2	1	5	6	7	4
11	3	6	5	7	2	4	1
12	5	4	1	3	6	7	2
13	5	2	1	4	3	6	7
14	2	3	1	4	5	6	7
15	1	2	3	6	7	5	4
16	2	3	1	7	6	4	5
17	1	3	2	5	7	6	4
18	2	3	1	6	7	5	4
